# A new frontier in CO_2_ flux measurements using a highly portable DIAL laser system

**DOI:** 10.1038/srep33834

**Published:** 2016-09-22

**Authors:** Manuel Queiβer, Domenico Granieri, Mike Burton

**Affiliations:** 1 School of Earth, Atmospheric and Environmental Sciences, University of Manchester, Oxford Road, Manchester M139PL, UK; 2 Istituto Nazionale di Geofisica e Vulcanologia (INGV), Sezione di Pisa, 50126 Pisa, Italy

## Abstract

Volcanic CO_2_ emissions play a key role in the geological carbon cycle, and monitoring of volcanic CO_2_ fluxes helps to forecast eruptions. The quantification of CO_2_ fluxes is challenging due to rapid dilution of magmatic CO_2_ in CO_2_-rich ambient air and the diffuse nature of many emissions, leading to large uncertainties in the global magmatic CO_2_ flux inventory. Here, we report measurements using a new DIAL laser remote sensing system for volcanic CO_2_ (CO_2_DIAL). Two sites in the volcanic zone of Campi Flegrei (Italy) were scanned, yielding CO_2_ path-amount profiles used to compute fluxes. Our results reveal a relatively high CO_2_ flux from Campi Flegrei, consistent with an increasing trend. Unlike previous methods, the CO_2_DIAL is able to measure integrated CO_2_ path-amounts at distances up to 2000 m using virtually any solid surface as a reflector, whilst also being highly portable. This opens a new frontier in quantification of geological and anthropogenic CO_2_ fluxes.

Quantifying CO_2_ emissions from geological and anthropogenic sources is a key objective for Earth and environmental scientists. The geological carbon cycle^1^ buffers the CO_2_ content of the atmosphere and thereby global temperatures, through a balance between CO_2_ emissions from volcanoes and geological sources, and CO_2_ take-up by temperature-dependent silicate rock weathering. This balance has maintained relatively stable atmospheric CO_2_ concentrations for the last 600 kyr^2^, thereby helping to stabilize temperatures on Earth^3^. Current estimates of global geological CO_2_ emissions have a large uncertainty^4^. The IPCC determine a geological CO_2_ flux of 370 Mt/yr^5^, while recent assessments of global CO_2_ flux indicate 540 Mt/yr^4^, neglecting potentially enormous but largely unquantified diffuse CO_2_ emission sources, such as that of the East African Rift, which alone may produce 71 Mt/yr^6^. Improving our quantitative understanding of geological CO_2_ emissions is therefore a fundamental goal for Earth scientists.

Geological CO_2_ emissions are the product of a complex exchange of CO_2_ between primordial mantle, mantle mixed with subducted material, continental crust and the exosphere. The degree to which CO_2_ emissions from arc volcanism reflects recycling of crustal CO_2_ or degassing of primordial mantle is an open question with large uncertainties^7^. Further measurements of the magnitudes of such fluxes can shed light on the CO_2_ recycling budget. Monitoring of gas emissions from volcanic areas is also useful for hazard assessment, as eruptive activity may be heralded by variations in gas composition and flux. CO_2_ is particularly useful as it is exsolved from magma at greater depths than other volatile species, and therefore can reflect deep processes^8^,^9^,^10^,^11^.

It is clear, therefore, that volcanic CO_2_ flux measurement is of great importance in the Earth sciences, however, performing this is by no means straightforward. To quantify gas fluxes, concentration profiles are needed. Owing to the often moderate magmatic CO_2_ signal compared with the ambient CO_2_ concentration volcanic CO_2_ fluxes are usually derived indirectly. *In-situ* point samples of CO_2_/SO_2_ ratios using MultiGAS^12^, or remote sensing gas composition measurement with COSPEC^13^,^14^,^15^,^16^, or open path FTIR spectrometry (OP-FTIR)^17^,^18^ are combined with measurements of SO_2_ flux using ultraviolet remote sensing^19^,^20^. CO_2_ fluxes may also be determined using airborne *in-situ* CO_2_ sensors and flying vertical grids through volcanic plume cross-sections^8^,^21^,^22^. Diffuse soil degassing CO_2_ efflux is typically measured *in situ* as well, using accumulation chambers^23^,^24^,^25^, LICOR analysers^26^ or the eddy covariance method^27^,^28^. Degassing of magmatic CO_2_ often occurs over large areas (10 s to 100 s of m). This includes vented and diffuse releases, such as soil leakage and volcano flank degassing^29^,^30^. Such fluxes are typically highly variable in space and time^31^, which poses a challenge to sporadically applied measurement techniques. Estimating gas fluxes from *in-situ* point measurements may thus lead to large uncertainties^32^. Moreover, *in situ* acquisitions require significant time and effort, especially for time-lapse studies. Even if they can be performed rather swiftly, such as by airborne sampling, *in situ* measurements often bear risk, e.g. when flying or hiking near volcanic craters.

Remote sensing techniques are usually safer to deploy than *in-situ* techniques and can offer attractive spatial coverage, yielding 1-D (path averaged) or 2-D concentration profiles from which more robust flux estimates can be retrieved. OP-FTIR^33^,^34^,^35^, hyperspectral imagers^36^,^37^ and differential absorption spectroscopy (DOAS)^38^,^39^ have all been used to measure magmatic gas compositions and fluxes. Passive remote sensing approaches, however, depend on light coming from the end of the measurement path. This may limit their flexibility, e.g. to clear sky conditions. Moreover, one risks to sense photons that have not penetrated the volcanic plume^40^.

Active remote sensing techniques, including open path laser absorption spectrometers^41^,^42^, carry their own light source and overcome these difficulties. Ideally, such instruments would not require designed retroreflectors, as this severely limits the flexibility of the measurement setup, in particular for airborne acquisitions. Active remote sensing approaches without the need for retroreflectors, such as LIDAR, overcome all previously mentioned drawbacks. Instruments based on differential absorption LIDAR (DIAL) have been performing pioneering measurements of SO_2_ at volcanoes over 20 years ago^43^,^44^, yet have not become a standard approach to measure volcanic gases. DIAL is increasingly used in global warming science to measure path averaged, ambient CO_2_ concentrations^45^,^46^,^47^,^48^ and has even been used to map a CO_2_ plume at an industrial site^49^. Recently, a range-resolving DIAL has successfully measured 2-D volcanic CO_2_ concentration profiles in the Campi Flegrei volcanic zone (Italy) from which CO_2_ fluxes have been inferred^50^.

Perhaps the main reasons why LIDAR based active remote sensing has not established itself as a standard for volcanic gas measurement is that LIDAR systems are not readily portable, due to their size and power requirements^46^,^47^,^49^, and require extensive operational resources (personal, expertise, setup time, logistics, maintenance)^44^,^47^. A versatile volcanology tool, instead, requires high portability and flexibility, since many volcanoes are in remote and difficult to access locations. Quick setup times and efficient data acquisition are also highly beneficial to minimise exposure to hazards. In the last decade, technology has moved sufficiently far forward to allow for the development of systems that fulfil these requirements.

We have developed an active remote sensing instrument based on the differential absorption principle, called CO_2_DIAL, which is specifically designed to detect volcanic CO_2_ emissions. This article presents results of an experiment in which the CO_2_DIAL was used to sense volcanic CO_2_ at the Solfatara crater and the Pisciarelli fumaroles in the active volcanic area of Campi Flegrei (CF) in Italy (Fig. 1a). CF is a nested caldera, resulting from two large collapses that occurred ~39 ka BP and ~15 ka BP^51^,^52^. Being close to the city of Naples, CF poses a direct threat to approximately one million people^53^. During the measurements at CF the CO_2_DIAL demonstrated its key capabilities, quickly acquiring profiles of CO_2_ path-amounts both near (~100 m) and far (~1 km) from the source after only ~15 min setup, for both diffuse and vented emissions, whilst being highly portable and operated by a single person. The retrieved CO_2_ fluxes from CF confirm the unusually strong degassing activity previously observed^26^,^54^.

These results demonstrate how the CO_2_DIAL could provide effortless monitoring of magmatic CO_2_ fluxes for eruption monitoring purposes at a volcanic area at unrest such as CF. The CO_2_DIAL is rugged and has been transported on passenger airplanes as ordinary carry-on luggage, which makes rapid international deployment uncomplicated. The CO_2_DIAL is platform independent and can, for instance, be operated airborne, e.g., from a helicopter, airplane and eventually a UAV. As such the CO_2_DIAL fills a key operational gap in volcanic CO_2_ sensing techniques and opens a new, exciting route to knowledge about volcanic systems, in particular regarding magma dynamics and the geological carbon cycle.

## Results

**Figure 1:**
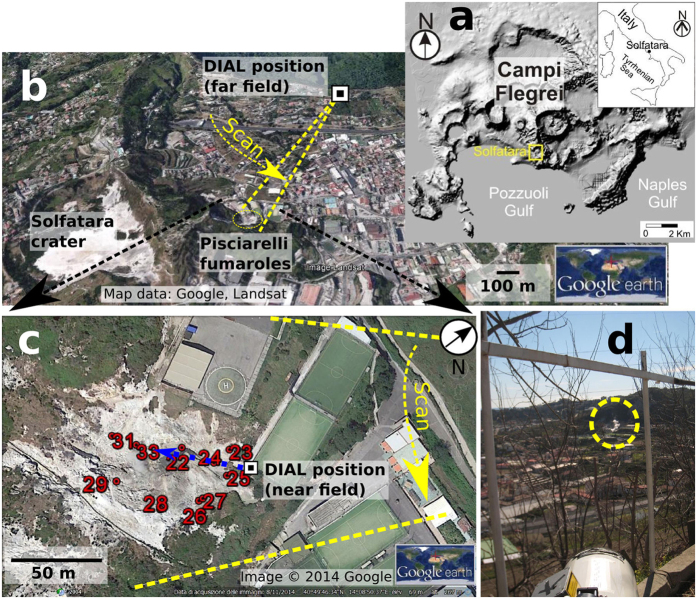
Geography of Campi Flegrei and overview of measurement sites. (**a**) Location of Campi Flegrei (CF) close to Naples in Italy. Both maps were obtained using the open-access digital elevation model of Italy, TINITALY/01^65^. (**b**) Overview of the measurement geometry of this study showing the Solfatara crater and the Pisciarelli fumaroles (within dotted circle). The angular extension of the scan for the measurement of the Pisciarelli fumaroles is indicated as well as the scan direction (arrow). (**c**) Close up nadir view of the Pisciarelli fumarole field depicting the numbered locations were CO_2_ concentrations have been measured *in situ* using a LICOR analyser. The blue dotted line marks the path of the fixed angle acquisition with length 55 m. (**d**) Photo taken from the DIAL position showing the telescope aligned with the Pisciarelli fumarole (dotted circle).

**Figure 2:**
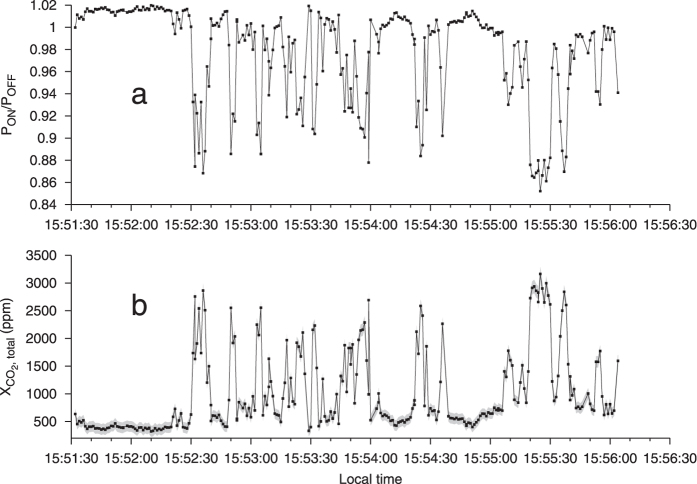
Near field static measurement at Pisciarelli. The range was 55 m. (**a**) Normalized intensity ratio (GR, Methods). Decrease indicates presence of excess CO_2_. The strong, pulse-like modulation of the GR is indicating the presence of large amounts of CO_2_. (**b**) Total path averaged CO_2_ concentrations obtained from CO_2_ path length concentration products (path-amounts) after dividing by the range (path length). At times they are over 3000 ppm. As in all of the following figures, the grey envelope depicts precision (1 SD, Equation (9) in Methods). Each point corresponds to 784 ms integration time.

The area of Pisciarelli encompasses a fault-related, fumarolic area, vigorously degassing water vapour and CO_2_, featuring a prominent, hot (90 to 110 °C) main vent and various smaller vents, including mud pools exhibiting gas bubbling. The Pisciarelli fumaroles therefore offer an optimal context for the CO_2_DIAL. The measurement geometry (Fig. 1b, see also Methods) mimics airborne acquisition and hence demonstrates the system’s capability for systematic airborne scanning of volcanic chains. The CO_2_DIAL was first placed in the vicinity of the Pisciarelli fumaroles (~60 m distance). During the measurement the optical axis of the instrument was pointing to the right of the centre of the plume (Fig. 1c) at a fixed angle. Strong, transient decreases in the grand ratio (GR, normalised intensity ratio, see Methods) are apparent (Fig. 2a), indicating the presence of considerable amounts of volcanic CO_2_ (Fig. 2b). The CO_2_ concentration at this angle was rapidly varying in a pulse like manner, which may be related to small scale wind eddies flushing in CO_2_ from a nearby vent. The path averaged CO_2_ concentrations (Equation (4), Methods) acquired with the CO_2_DIAL (between ~400 and 3163 ppm) agree well with those measured *in situ* during the acquisition of the data using a LICOR CO_2_ analyser (between 650 and 3000 ppm along the CO_2_DIAL path). Note that, in the following, CO_2_ concentrations measured by the CO_2_ DIAL always refer to path averaged concentrations. We found that working at a small distance between the instrument and the plume was impractical, since, due to the limited radial scanning velocity, scans took too much time. Depending on local, small-scale wind eddies in the plume area, opaque, condensed water vapour occasionally covered the measurement path. Sunlight scattered by these cloud then saturated the detector and made a repetition of the scan necessary. By moving further away we were able to perform a more rapid scanning of the plume area, reducing the chance of scattered sunlight interfering with the scan.

**Figure 3:**
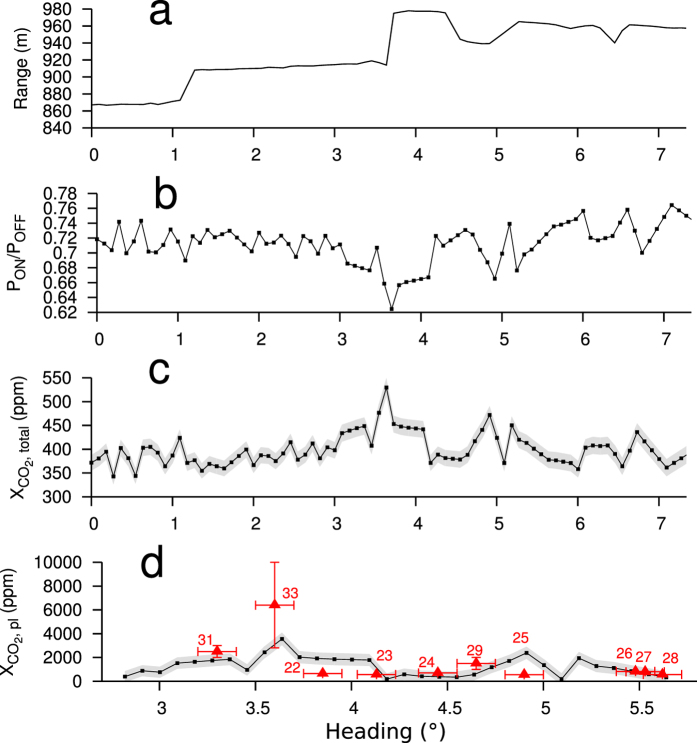
Example of far field scan at Pisciarelli. (**a**) Range measurements versus scan angle (heading). The ranges were measured using a range finder LIDAR (Methods). They defined the path length. (**b**) Grand ratio (GR) versus heading. (**c**) Total path averaged CO_2_ concentration. Also shown is the measurement precision (1 SD) in grey. (**d**) In-plume CO_2_ concentrations. They were derived from data in c) using Equation 4 (Methods). The numbered triangles depict the values and ranges as well as lateral position uncertainties of the *in situ* measurements (Fig. 1c). Note that these have been acquired ~20h earlier. Thus, they serve as approximate reference only. Moreover, concentrations in d) from the CO_2_DIAL represent path averages with contributions from across the plume, while the *in situ* values show concentrations measured at a single point in the plume.

The CO_2_DIAL was located in a small parking lot located at an exposed part of a slope ~940 m northeast of Pisciarelli (Fig. 1b,d). The telescope was visually aligned with the Pisciarelli plume by means of a 90° flip mirror in the ocular tube of the telescope. The angular coverage of the scans was chosen generously at 7.5°, in order to include surroundings free of plume CO_2_ into the scan, to help unambiguously identify the plume. The optical power was set to 1 W (66% of maximum). Scans were performed by means of a step motor that pivoted the complete transmitter and telescope unit around the region of interest (Fig. 3a). The angular velocity of the scan was fixed at 0.525 mrad/s. A single scan took ~4 min out of which the plume itself was traversed in ~100 s. The CO_2_DIAL detected a strong increase in column integrated CO_2_ path length concentration product (hereafter called CO_2_ path-amount, Equation (3) in Methods). The latter varied from ~4 × 10^5^ ppm.m up to 4.8 × 10^5^ ppm.m near the centre of the plume, which, after dividing by the target range (measured by an onboard laser range-finder), corresponds to a path averaged concentration of up to 530 ppm (Fig. 3c). Assuming that this excess CO_2_ was contained entirely in the plume, and estimating the plume diameter, permits us to estimate the in-plume concentration of CO_2_ as 3600 ± 530 ppm (1 SD, see Equation (5) in methods for derivation of the in-plume concentration), in good agreement with the LICOR *in situ* data (Fig. 3d).

**Figure 4:**
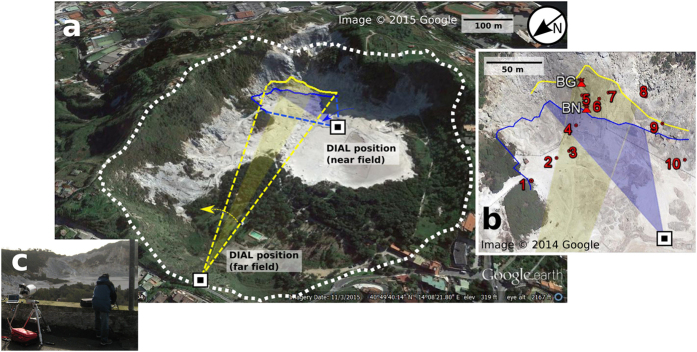
Overview of the measurement geometry at Solfatara. (**a**) Solfatara crater. The crater area is marked by the white dotted line. The extension of the far and near field scans and scan is indicated by dotted lines. The curved arrows mark the scan direction. Shaded areas depict regions of interest, i.e. the sectors for which flux results are reported in Table 1 (blue near field, yellow far field). The target distances versus heading are indicated for the near and far field scans (blue and yellow lines). (**b**) Nadir photo showing a zoom around the main fumaroles. Shown are the *in situ* measurement points and the locations of the main fumaroles Bocca Nuova (BN) and Bocca Grande (BG). (**c**) Photo of the CO_2_DIAL while measuring from the far field position.

**Figure 5:**
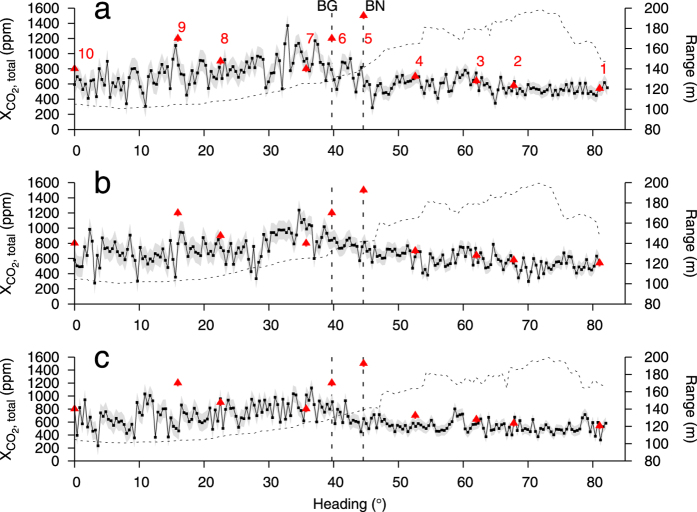
Time lapse near field scans across the diffuse degassing area in the Solfatara crater. See also Fig. 4b. The dashed curve shows the measured range per heading. The headings corresponding to the positions of BN and BG are indicated. Since the acquisition was done within the degassing area, the total concentrations instead of the in-plume concentrations are shown. The triangles depict the LICOR *in situ* values acquired during acquisition of these scans, identified by the numbers (Fig. 4b). All three scans were carried out on March 3, 2016. (**a**) Scan performed between 11:31:39 and 11:43:06. (**b**) Scan performed between 11:52:48 and 12:04:27. (**c**) Scan performed between 12:07:20 and 12:19:05. The short scanning interval makes it possible to resolve fluctuations in the CO_2_ concentration on a minute scale. By comparing the magnitude of the main peak near 35° it becomes evident that at places the average CO_2_ concentration fluctuated by more than 20%.

Solfatara is a tuff cone associated with intense hydrothermal activity, manifesting fumaroles, hot soils and diffuse CO_2_ degassing. The Solfatara crater therefore offered an excellent possibility to probe a mixture between fumarole and diffuse degassing. An overview of the setup is given in Fig. 4a. The scanning angular velocity was 2.1 mrad/s. The two main fumarole vents, Bocca Nuova (BN) and Bocca Grande (BG), are located near the edge of the crater (Fig. 4b). *In-situ* measurements using the LICOR analyser that took place during the CO_2_DIAL scans confirmed high CO_2_ concentrations in their vicinity, as expected. Solfatara furthermore features zones of weaker degassing strength and an extensive diffuse soil CO_2_ output. The corresponding CO_2_ concentrations were clearly detectable with the LICOR analyser. A relatively high scanning inclination angle would have been necessary to scan over the main vents. This would, however, likely have missed excess CO_2_ concentrations in the centre of the crater (towards the instrument location) and further south along the rim (*in situ* points 1 to 4), in particular the weaker zones of anomalous CO_2_ release, both vented and diffuse. In addition, since we could visually observe that the plume water vapour movement was a superposition of vertical flow and a horizontal component towards south/southeast, it was clear that the plume CO_2_ would be advected away from BN and BG towards the southeast (roughly along the crater rim). We therefore chose a scanning inclination such that the scan altitude at BN was 2 m above the ground, which while missing the direct observation of the BG vent, would capture the CO_2_ emissions from the BG vent downwind. From Fig. 5 it can be seen that the CO_2_DIAL detected high CO_2_ concentrations near 35° that are compatible with the LICOR *in situ* values, both having a symmetric profile. However, with respect to the *in situ* values these CO_2_ concentrations are shifted by 9°, i.e. ca. 20 m further south. This can be explained by the fact that the *in situ* measurements at points 5 to 8 were performed at slightly higher elevation (Fig. 4b) and were not covered by the CO_2_DIAL measurement path during the near field scans. At 60° another zone of higher CO_2_ concentrations can be identified in all three scans (Fig. 5a–c), which reveals the presence of a diffuse CO_2_ source. Given the associated position, the increase in CO_2_ concentration near 50° (Fig. 5a–c) could be related to BC, a vent that appeared recently^26^. Lastly, there are numerous, persistent small-scale fluctuations in the profiles (Fig. 5a–c), such as near 16°, confirming widespread CO_2_ degassing activity.

**Figure 6:**
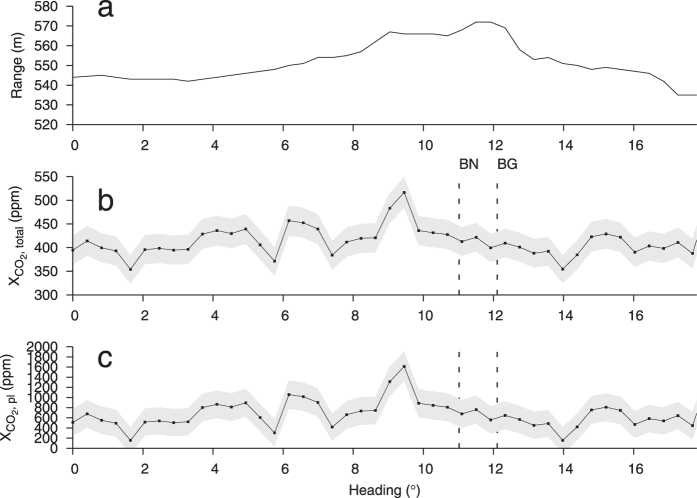
Result of a far field scan across the area around BN and BG at Solfatara crater. The scan was performed between 18:32:29 and 18:35:13 on March 2, 2016, i.e. ca. 18 h prior to the acquisition in Fig. 5. (**a**) Measured ranges per heading. (**b**) Total path averaged CO_2_ concentrations. (**c**) Average in-plume CO_2_ concentrations. The heading of the peak near 9.5° corresponds to 35° in Fig. 5.

To further test the results from the CO_2_LIDAR, we scanned the same degassing feature from a location further away, with the instrument installed ~550 m northwest of the main vents (Fig. 4a). The inclination for these measurements included both BN and BG (Fig. 4b). The detected CO_2_ concentration versus the heading angle appears smoother (Fig. 6). This could be partly due to the slightly higher inclination (beam travelled ~5 m above ground near BN) and larger atmospheric contribution which both tend to diminishes the relative contribution of CO_2_ from weaker vents. Moreover, using the same angular scan velocity of 2.1 mrad/s as before the probed sector size was ~1.2 m/s compared with 30 cm/s for the near field scan. As a result, the effect of the CO_2_ is averaged, contributing to a smoother profile. Out of all scans performed from the Solfatara far field position, the maximum in-plume volcanic CO_2_ concentration detected with the CO_2_DIAL was 1900 ± 303 ppm.

### CO_2_ fluxes

Our CO_2_ path-amount profiles together with estimated vertical plume transport speeds were used to retrieve CO_2_ fluxes as described in the Methods section. Table 1 summarizes the results for the scans carried out at Pisciarelli and Solfatara during the complete duration of the experiment. For Solfatara the sectors considered for flux retrieval include the area around the main vents as shown in Fig. 4b. As already suggested by the CO_2_ concentrations (Fig. 5), the CO_2_ fluxes vary strongly during the course of even tens of minutes (Table 1). The time-averaged CO_2_ flux estimated for Pisciarelli is 3.1 ± 2.5 kg/s (1 SD) or 266 ± 212 tons/day. The average over subsequent scans yields a mean CO_2_ flux for Solfatara of 9.3 ± 4.7 kg/s (805 ± 408 tons/day) for the near field and 7.7 ± 4.5 kg/s (664 ± 386 tons/day) for the far field scans, associated with a total average of 715 ± 394 tons/day for Solfatara.

## Discussion

We have used the newly developed CO_2_DIAL to sense volcanic CO_2_ at the restless Campi Flegrei volcanic zone in Italy. The CO_2_ concentrations measured with the CO_2_DIAL are in good agreement with *in situ* values acquired with a LICOR CO_2_ analyser. Care has to be taken when comparing *in situ* values with those from the CO_2_DIAL, since the *in situ* measurements detect contributions from a point-like volume around the instrument, whereas concentrations detected by the CO_2_DIAL correspond to contributions from points all along the column between instrument and hard target (Methods). The most robust comparison is produced when the CO_2_ concentration in the column is governed by the same CO_2_ source sensed by the *in situ* device. For Solfatara, this was the more the case for near field scans (Fig. 5) than for the far field scans (Fig. 6).

CO_2_ path-amount profiles have been converted into fluxes using the vertical plume transport speed estimated from video footage (Methods). The precision of the CO_2_ concentration along with error of the vertical CO_2_ transport speed propagate to an error of the CO_2_ flux (Equation (12), Methods), which is on average 60% (mean over relative errors of all computed fluxes). The system has yet not reached its full potential, mainly in terms of precision. Future work will focus on increasing precision to be able to sense more subtle volcanic CO_2_ signals and increase confidence of the retrieved CO_2_ fluxes. Further, the plume speed measurement technique will be improved to reduce the related uncertainty.

While much variability in the measured CO_2_ path amounts is the product of local fluctuations in transport speed and direction within the plume, the varying CO_2_ path-amount detected at Pisciarelli (Fig. 2) may reflect a varying fumarole degassing rate on a timescale of tens of seconds^42^. A further, more systematic investigation, including a frequency analysis, would enable improved identification of the origin of the variability. This represents one of the doors opened by new active remote sensing tools with high temporal resolution, such as the CO_2_DIAL. A longer time-scale variability in CO_2_ flux is observed at both Pisciarelli and Solfatara. Table 1 suggests that strong variations of the CO_2_ flux by 20 to 76% may occur within less than an hour, which is consistent with previous observations^50^ which found average degassing rates at Pisciarelli of 227 tons/day (mean of all measurements) in late 2014. That result is slightly lower than the average of 266 ± 212 tons/day found in this study. Results from more previous years are significantly lower (e.g., ~180 tons/day in 2012^42^). The high quality dataset of CO_2_ fluxes captured by the Osservatorio Veusiviano geochemistry team has revealed that fluxes have risen by ~40% every year between 2008 and 2014^55^. The present finding suggests an increase in CO_2_ flux by a factor of 266/227 (17%) in 1.5 years, which is reasonably consistent with previous observations, and certainly indicates a general trend of increasing CO_2_ efflux from Pisciarelli. This contributes to the general picture of increased degassing associated with enhanced ground uplift observed in the area^26^,^54^.

For the mixed degassing (vented and diffuse) at Solfatara it is more challenging to compare with *in-situ* fluxes as the boundaries of the area investigated are generally not exactly the same. This is already the case for the near and far field Solfatara scans of the present study, which span different areas. Yet, the CO_2_ fluxes for the far and the near field agree within error. Note that the CO_2_DIAL measures path-amounts, (Methods) with contributions from vented and diffuse CO_2_ emissions all across the measurement line. Therefore, differences between the fluxes from the far and near field measurement are very likely due to the different integrated soil areas covered by the near and the far field scans. The average flux of 715 tons/day found in this study is considerably larger than CO_2_ fluxes from previous measurements between 2012 and 2013 at the main vents (BN and BG), which yielded between 251 and 300 tons/day^26^,^42^. However, these values account for the fumarole degassing, excluding diffuse degassing. The fumarole emissions near BG and BN were estimated to correspond to roughly 40% of the total soil CO_2_ output^26^, resulting in a total flux of ~1560 tons/day, however, for the whole Solfatara/ Pisciarelli area. In line with this value, a diffuse degassing rate of ~1500 tons/day for a 0.5 km^2^ area of Solfatara has been reported previously^56^. It is hard to scale the calculated flux in this study up to the size of the whole degassing area of CF as there are areas with a very low flux and areas with a very high flux. We can, however, provide an estimation for the Solfatara crater alone, excluding the area of the Pisciarelli fumaroles. For the Solfatara far field scans, the sector accounted for in the flux computation of this work corresponds to a surface of ~20000 m^2^ inside the Solfatara crater (region of interest, shaded area in Fig. 4a,b). When integrating over all headings (dashed triangle, Fig. 4a), as opposed to the region of interest only, the flux doubles on average, amounting to ~1400 tons/day. This larger region has an area of ~43000 m^2^, which corresponds to ~14% of the crater area of ~300000 m^2^, approximately bounded by the dashed white line in Fig. 4a. Flux maps of previous *in situ* studies^57^,^58^ suggest that the area covered in our study accounts for 31% (=43000/140000) to 61% (=43000/70000) of the anomalous degassing area inside the crater. Assuming a consistent flux per unit area throughout the crater implies a CO_2_ flux between 2300 and 4600 t/day for the Solfatara crater alone, including the contribution of the main fumaroles (BN and BG) and other minor vented emissions on the southeast wall of the crater. In the future, a more spatially comprehensive flux measurement is envisaged, scanning the whole of Solfatara cater, which will allow to compare fluxes of the Solfatara area with greater confidence.

Whilst smaller than at Pisciarelli, Solfatara has seen a steady increase in CO_2_ degassing rate in the last decade or so^55^. Recent findings^59^,^60^ indicate that apart from the vented degassing area near BN and BG there is a “hot spot” zone of approximately rectangular shape along the south-eastern edge of the Solfatara crater (between 0 and 36° in Fig. 5), featuring high anomalous CO_2_ release, in line with the LICOR data of this study. It underpins the high CO_2_ concentrations detected in that zone by the CO_2_DIAL and furthermore supports that the associated CO_2_ amounts largely correspond to diffuse emissions and minor vents with formerly unknown CO_2_ output.

In summary, the CO_2_ fluxes found with the CO_2_DIAL are compatible with previous results, indicating strong, increasing CO_2_ degassing at CF. To the best of our knowledge, it is the first time that a man-portable active remote sensing system has been used to acquire volcanic CO_2_ profiles. The outcomes show the tremendous potential of a portable gas remote sensing system. The ease and flexibility with which the presented data has been attained represents a breakthrough in the measurement of volcanic CO_2_. The results show that the CO_2_DIAL is a powerful tool to overcome the aforementioned drawbacks of *in situ* measurement techniques by offering a faster, safer and comprehensive acquisition (spatial coverage yields CO_2_ concentration profiles). The platform demonstrated its capability to execute effortless time-lapse acquisitions within minutes or tens of minutes, depending on the area covered and the target range. This is a very desirable feature to analyse correlation between CO_2_ degassing and uplift at inhabited volcanic zones, such as CF, where days can be a crucial time scale.

In conclusion, a man-portable active remote sensing tool as the CO_2_DIAL represents a step change in volcanology by opening completely new measurement possibilities. One of these prospects is uncomplicated, systematic probing of arc volcanoes to populate the global volcanic CO_2_ flux data inventory and help constraining the geological carbon cycle and deliver new insights in the magmatic plumbing system of subaerial volcanoes. Applications of the CO_2_DIAL also include more general areas of environmental and Earth system science.

## Methods

### DIAL principle

The CO_2_DIAL is based on continuous wave, hard target differential absorption LIDAR (DIAL), measuring path averaged CO_2_ concentrations. A DIAL transmits and receives photons at two wavelengths, one corresponding to a rotational-vibrational transition of CO_2_ where photons are being absorbed (*λ*_*ON*_) and one where there is no associated absorption (*λ*_*OFF*_). *λ*_*ON*_ and *λ*_*OFF*_ are closely spaced to each other so that atmospheric propagation effects other than absorption by the probed molecule can assumed to be negligible. By taking the ratio of the optical powers associated with the received signals for *λ*_*ON*_ and *λ*_*OFF*_ one arrives at^47^


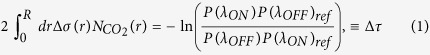


where *R* is distance between the instrument and the hard target (e.g. the soil), Δ*σ* is the difference between the molecular absorption cross sections of CO_2_ associated with *λ*_*ON*_ and *λ*_*OFF*_, *P*(*λ*) is the received power (hereafter referred to as science signal as it carries the information of scientific interest) and *P*(*λ*)_*ref*_ is the transmitted optical power (referred to as reference signal). The latter is measured as a reference to normalize fluctuations of the transmitted power. Δ*τ* is the differential optical depth. The ratio of the normalized powers in Equation (1) is referred to as the grand ratio (GR). The presence of excess CO_2_ leads to a decrease in GR (Fig. 2a). By measuring the GR as well as *R* one may obtain the path averaged CO_2_ number density 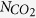, which for a horizontal measurement path (as in this work) can be converted to a concentration (in ppm) as


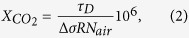


where *N*_*air*_ is the (moist) air number density computed with knowledge of specific humidity, air pressure and temperature.

### The CO_2_DIAL

Supplementary Fig. S1 presents an overview of the CO_2_DIAL instrument as used for this work, which is an all-fiber optic instrument. The instrument is a prototype with development costs of the order of 100 k €. A next generation commercial prototype is planned with associated costs roughly a third of that. The CO_2_DIAL is designed to measure path averaged CO_2_ concentrations for path lengths up to 2000 m. The instrument has been developed to acquire volcanic CO_2_ concentration profiles from which CO_2_ fluxes can be deduced. The complete system weighs ~26 kg and uses a power of ~20 W in idle mode and ~70 W when the laser is emitting at its full optical power. The CO_2_DIAL can operate for ~1 h on a single 600 g, 5000 mAh lithium polymer battery. The laser consists of two continuous wave distributed feedback (DFB) fiber seed lasers emitting at *λ*_*ON*_ = 1572.992 nm and *λ*_*OFF*_ = 1573.173 nm^61^. The seed laser beams are amplitude modulated using two LiNbO_3_ electro-optical modulators (EOM) at a respective sine tone near 5 kHz and simultaneously amplified by an Erbium doped fiber amplifier (EDFA) before being transmitted. Light backscattered by a hard target is received by a commercial 200 mm diameter Schmidt-Cassegrain Telescope with a focal length of 1950 mm. The analogue to digital converter (ADC) operates at 250 kSamples s^−1^ and has a resolution of 16-bit. The integration time per scan angle was set to 4000 EOM modulation periods, which corresponds to chunks of length 784 ms for both science and reference channel. Each of these chunks of data is demodulated using a digital lock-in routine (or through performing a FFT for diagnostic purposes) written in National Instruments LabVIEW. This is done in real-time for quality control purposes (e.g. to check for abnormal baseline drift) and again in the post processing of the data to compute CO_2_ concentrations. After the lock-in operation one arrives at four DC signals associated with *P*(*λ*_*ON*_), *P*(*λ*_*OFF*_), *P*(*λ*_*ON*_)_*ref*_ and *P*(*λ*_*OFF*_)_*ref*_. The GR and Δ*τ* are calculated using the right hand side of Equation (1) after taking the mean of each of the four signals. Each point in Figs 2, 3, 5 and 6 is associated with an integration time of 784 ms. The target distance *R* is measured by a range finder (DLEM, Jenoptik, Germany), based on a 1550 nm LIDAR with pulse energy of 500 μJ and accuracy <1 m. The working principle of the range finder is the time of flight measurement. The integration time was set to 500 ms.

### CO_2_ concentration retrieval

DIAL instruments generally have an offset, i.e. Δ*τ*(*R* = 0) ≠ 0. In addition, due to the fact that the metastable state lifetime of the erbium atoms is comparable to the period of the seed laser amplitude modulation, the EDFA distorts the amplitudes of the seed laser signals. Demodulation of the distorted science and reference signals creates an offset that depends on the signal power, but which is repeatable. As a result, the measured GR are shifted with respect to those expected from Equation (1). Therefore, to retrieve path averaged CO_2_ concentrations a calibration is performed using the ambient CO_2_ at the measurement site, away from any CO_2_ degassing (Supplementary Fig. S2). To this end, GR were measured in the well-mixed ambient atmosphere for various ranges, converted to differential optical depths using Equation (1) and plotted against the CO_2_ path length concentration product (path-amount, product of ambient CO_2_ concentrations and ranges). A straight line,


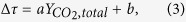


was fitted to the ensemble of points using a least square algorithm. Equation (3) was used as the calibration curve, with slope *a* (in ppm.m^−1^) and instrumental off set *b*. Employing the calibration curve the measured Δ*τ*at CF were converted to CO_2_ path-amounts 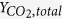 (in ppm.m), which were used for further computations as detailed below.

### Conversion of CO_2_ path-amounts to in-plume CO_2_ concentrations

**Figure 7:**
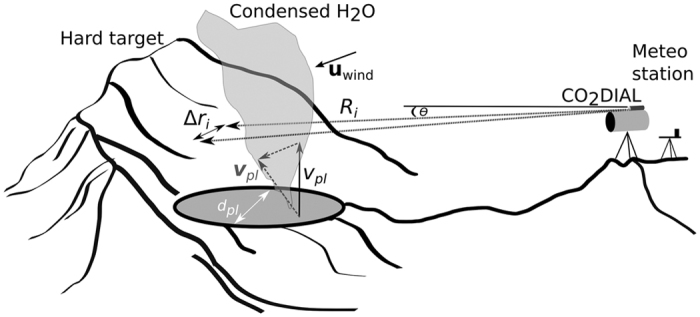
Sketch of a typical measurement geometry. The hard target can be any surface. The transmitter/telescope unit is pivoted around the degassing feature (lateral extension 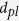, Equation 7), *R*_*i*_depicts the range, Δ*r*_*i*_ the lateral distance increment (in m) at angular step *i*. The wind is indicated as a velocity vector u_*wind*_ (in bold). The vertical plume transport speed v_*pl*_ is shown here as a vector resulting from superposition of the inherent velocity of the ejected CO_2_ and the wind speed. For the flux computation the magnitude of its vertical component v_*pl*_ is needed (also shown in the figure).

Figure 7 shows a simplified sketch of a typical measurement geometry at Campi Flegrei. Since the CO_2_DIAL yields CO_2_ path-amounts (ppm.m), volume CO_2_ concentrations measured *in situ* (in the plume area) cannot be compared directly with the result from the CO_2_DIAL. If the plume extension approximately equals the CO_2_DIAL path length, dividing column amounts by the path length may be done, i.e.


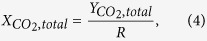


such as shown in Figs 2b and 5, yielding path averaged CO_2_ concentrations. Of course, for path lengths much longer than the plume extension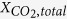 cannot be compared with *in situ* values taken in the plume. In these cases CO_2_ column amounts were converted to in-plume concentrations 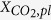, which account for the volcanic and the ambient CO_2_ (such as detected by the LICOR analyser). They were obtained using


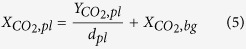


where 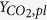 is the volcanic excess CO_2_ path-amount (in ppm.m), obtained as


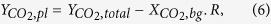


where 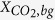 = 380 ppm (ambient CO_2_ concentration at CF from *in situ* measurement) and *R* is the target range.*d*_*pl*_ is the lateral dimension of the plume, calculated as


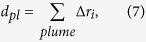


where Δ*r*_*i*_ is the lateral distance increment (in m) per angular step *i*. 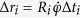 with the range per angle *R*_*i*_ (see Fig. 7), the angular scan velocity 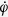 and the time interval Δ*t*_*i*_ between points (angles), retrieved from the time stamps of the data chunks.

### Identifying the plume

An increase in volcanic CO_2_ would lead to an increased column averaged amount of CO_2_ and would thus decrease the GR (increase Δ*τ*). When performing a scan across a sufficiently strong CO_2_ emission this behaviour occurs as a function of scanning angle. By repeatedly scanning across the emission feature, artefacts can be ruled out and the decrease in GR as well as the angle where this happens can be unambiguously attributed to CO_2_ originating from anomalous degassing activity. Of course, this assumes that the plume extension is sufficiently stable between subsequent scans. For vented degassing, the position of the telescope (and hence the angle) when it entered an anomalously degassing zone could be determined visually using the telescope. This procedure works well when the scanned horizon also contains areas with ambient atmosphere only. This was not the case for the near field Solfatara scans since the instrument was placed inside the anomalous degassing (i.e. the main plume) itself already. Nonetheless, one could identify a rather stable symmetric increase in CO_2_ concentration, which was chosen as the region of interest to integrate over to arrive at the CO_2_ flux.

### Flux retrieval

To compute the mass flux of CO_2_ (in kg/s) the volcanic excess CO_2_ path-amounts (Equation 6) were integrated over the lateral plume extension as


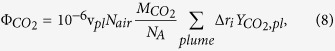


where v_*pl*_ is the plume transport speed (m/s), more precisely the component of the transport speed vector perpendicular to the scanned cross section. It was assumed constant during the scan and across the plume cross sectional area (see section plume speed estimation). Since the scans were performed horizontally, here it refers to the magnitude of the vertical component of the transport speed vector (Fig. 7). *N*_*air*_ is the number density of moist air, computed using meteorological data (pressure, temperature, humidity) acquired by a portable meteo station close to the instrument (Fig. 7). 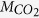 is the molar mass of CO_2_ (kg mol^−1^) and *N*_*A*_ is Avogadro’s constant (mol^−1^). For a vented emission, as at Pisciarelli, the integration boundaries were determined by the angles between which there was a steady increase followed by steady decrease in CO_2_ concentration above the background. For Solfatara, the integration was carried out for the region of interest around the main vents, that is, between *in situ* measurement points 8 and 4 (near field) and 8 and 2 (far field) (Fig. 4a,b, and section identifying the plume). This interval was chosen since the scans indicated that it contained the CO_2_ from the main fumaroles as well as CO_2_ from along the crater edge between *in situ* point 5 and 8, discernible by a repeatable feature (Fig. 5).

### Plume speed estimation

v_*pl*_ may be estimated from the wind vector. However, particularly close to the ground or close to reliefs, such as at Solfatara, the topography gives rise to a complicated wind vector field. The wind speed may thus be quite different from the actual transport speed of the plume. Further, the meteo station was not measuring the wind field components, but the true wind direction. For the near field Solfatara measurements the wind veered around 285° (mean of wind speeds for all scans). For the Solfatara far field measurement on 02/03/2016 there was no wind direction data available as the compass was not calibrated. For the Solfatara far field measurements on 03/02/2016 the mean wind direction was 168°. For the scans at Pisciarelli the wind direction measured was on average 200°, which means the Pisciarelli fumaroles were in the wind shadow of the Solfatara crater rim. v_*pl*_ was estimated therefore directly from the plume movement. It was assumed that the movement of the visible condensed plume water vapour aerosol propagated with the same velocity as the volcanic CO_2_. Video footage with a resolution of 480 × 640 pixels^2^ was acquired from the plume using a commercial digital camera placed next to the CO_2_DIAL telescope. During each set of scans a video clip of the plume was produced (4 in total). A video analysis program (Tracker from Open Source Physics) was used to analyse the video footage. At a given video frame a pixel of the plume was picked (Supplementary Fig. S3a). The pixel (which corresponds to a parcel of gas) was moving with each frame until it eventually experienced a decrease in brightness, usually after 3 to 13 seconds, due to associated water droplets evaporating. The analysis focused on pixels within and near the telescope field of view. Transformation from pixel into real world coordinates was carried out via distance calibration using the size of a real world feature (e.g. a house). These features were chosen such that they were aligned approximately parallel to the plane of the plume. In cases where these features were not perpendicular to the telescope line of sight (for Pisciarelli) their projected size was used, employing Google Earth software. The camera line of sight was usually quasi perpendicular to the (on average vertical) plume transport direction (for Pisciarelli, cos *θ* = 0.997 in Fig. 7). For the Solfatara scans (both near and far field) the plume speed had a horizontal component, which was, however, quasi perpendicular to the telescope viewing direction. This resulted therefore in a negligible length projection error. Frames were converted to time using the frame rate of the camera (30 Hz). The tracking software enabled to extract the relative vertical position of a pixel (y-position). v_*pl*_ was derived from the slope of the regression line fitted on the y-positions (Supplementary Fig. S3b). The error was extracted from the root mean square error of the fit (RMSE of observed minus modelled y-coordinates). For a tracked parcel of gas this error accounts for fluctuations in transport speed due to a complex, perhaps turbulent wind field. Note that v_*pl*_ derived in that fashion represents an average speed over the course of the measurement. The RMSE represents an uncertainty over time, but for only a part of the plume. The procedure was therefore repeated for various parcels across the plume. The retrieved speeds and their RMSEs are summarized in Supplementary Table S1. Comparing the derived speeds provided a way to assess fluctuations in vertical transport speed across the plume as viewed by the CO_2_DIAL telescope. Assuming normally distributed plume speeds the associated standard deviation was computed using Student’s t distribution^62^, developed for small number of samples, which are, however, statistically meaningful. Here, 8 tracks have been performed per video (Supplementary Table S1). Note that each of the 8 plume speeds per video is based on a fit of >100 points and thus exhibit a certain robustness. The uncertaintyΔv_*pl*_ of v_*pl*_ was estimated as the maximum out of the Student’s SD and the mean of the RMSEs (Supplementary Table S1). For the scans carried out at far field Solfatara at the evening of 02/03/2016, y-positions versus time were usually quite steady, suggesting a rather laminar plume flow, which resulted in a lower RMSE (Supplementary Fig. S3b and Table S1). Supplementary Fig. S3c,d show two less steady y-tracks of parcels of the far field Solfatara scans the morning after, suggesting a higher degree of turbulence and hence a higher associated uncertainty of the plume transport speed.

### Estimating measurement precision

The total relative uncertainty of the CO_2_ path-amount was evaluated as


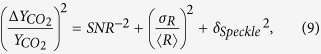


with the signal-to-noise-ratio (SNR)


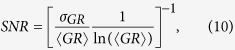


where 〈*GR*〉 and *σ*_*GR*_ are the mean and standard deviation of the grand ratio values, respectively. They were estimated from time series acquired at fixed angles in between the scans at CF. The SNR accounts for all noise sources occurring during acquisition, including instrumental noise, non-stationary baseline drift, solar background noise, atmospheric noise (mostly air turbulence) and perturbation by aerosol scattering. It is therefore an indicator of the instrument’s true measurement performance in the field^63^. The second term quantifies the relative range error (standard deviation of ranges *σ*_*R*_ over mean of ranges 〈*R*〉), which is typically ~1 m. The relative uncertainty due to hard target speckle was estimated as^64^


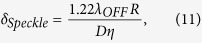


where *D* is the spot diameter on the hard target (in m) and *η* is the dimension of the telescope FOV (in m) on the hard target.

The total relative error of the CO_2_ flux was computed as


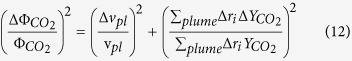


## Additional Information

**How to cite this article**: Queiβer, M. *et al*. A new frontier in CO_2_ flux measurements using a highly portable DIAL laser system. *Sci. Rep.* **6**, 33834; doi: 10.1038/srep33834 (2016).

## Supplementary Material

Supplementary Information

## Figures and Tables

**Table 1:** Results of selected scans performed at Solfatara and Pisciarelli.

Acquisition date and time	Transport speed v_*pl*_ (m/s)	Flux 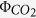 (kg/s)
	**Far field Solfatara**	
02/03/2016 18:11	1. 53 ± 0.5	5.8 ± 3.5
02/03/2016 18:19	1. 53 ± 0.5	7.9 ± 4.0
02/03/2016 18:29	1. 53 ± 0.5	5.9 ± 3.5
**02/03/2016 18:32**	1. 53 ± 0.5	5.1 ± 3.4
02/03/2016 18:38	1. 53 ± 0.5	6.4 ± 3.5
03/03/2016 10:19	3.12 ± 1.1	12.5 ± 6.4
03/03/2016 10:24	3.12 ± 1.1	10.2 ± 7.1
**Mean** ± **1 SD**		**7. 7** ± **4.5** (**664** ± **386** tons/day)
	**Near field Solfatara**	
**03/03/2016 11:31**	1.68 ± 0.6	11.7 ± 5.8
**03/03/2016 11:52**	1.68 ± 0.6	10.7 ± 5.1
**03/03/2016 12:07**	1.68 ± 0.6	8.1 ± 4.4
03/03/2016 12:39	1.68 ± 0.6	6.8 ± 3.6
**Mean** ± **1 SD**		**9.3** ± **4.7** (**805** ± **408** tons/day)
	**Far field Pisciarelli**	
04/03/2016 13:18	1.69 ± 0.8	2.1 ± 2.2
**04/03/2016 13:33**	1.69 ± 0.8	3.6 ± 2.4
04/03/2016 13:50	1.69 ± 0.8	3.6 ± 2.7
**Mean** ± **1 SD**		**3.1** ± **2.5** (**266** ± **212** tons/day)

The experiment lasted between 02/03/2016 evening and 04/03/2016 afternoon. Shown are the vertical plume transport speeds as derived from video analysis and the values of the CO_2_ fluxes 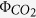. Scans presented in the figures have their time marked in bold. For details on the error calculation please see Methods.
