# Supplementary material for: A new frontier in CO2 flux measurements using a highly portable DIAL laser system
Source: Sci Rep. 2016 Sep 22;6:33834. doi: 10.1038/srep33834 (PMC5031984; doi:10.1038/srep33834)
Supplement: Supplementary Information [file srep33834-s1.pdf]

A new frontier in CO_2 flux measurements using a highly portable DIAL laser system

Manuel Queißer[1,*], Domenico Granieri[2], Mike Burton[1]

[1,*]School of Earth, Atmospheric and Environmental Sciences, University of Manchester, Oxford Road, Manchester M139PL, UK, Email: manuel.queisser@manchester.ac.uk, Tel.: +44(0)161 2750778, Fax.: +44(0)161 306 9361

[2]Istituto Nazionale di Geofisica e Vulcanologia (INGV), Sezione di Pisa, 50126 Pisa, Italy, Email: domenico.granieri@ingv.it

[1]School of Earth, Atmospheric and Environmental Sciences, University of Manchester, Oxford Road, Manchester M139PL, UK, Email: mike.burton@manchester.ac.uk

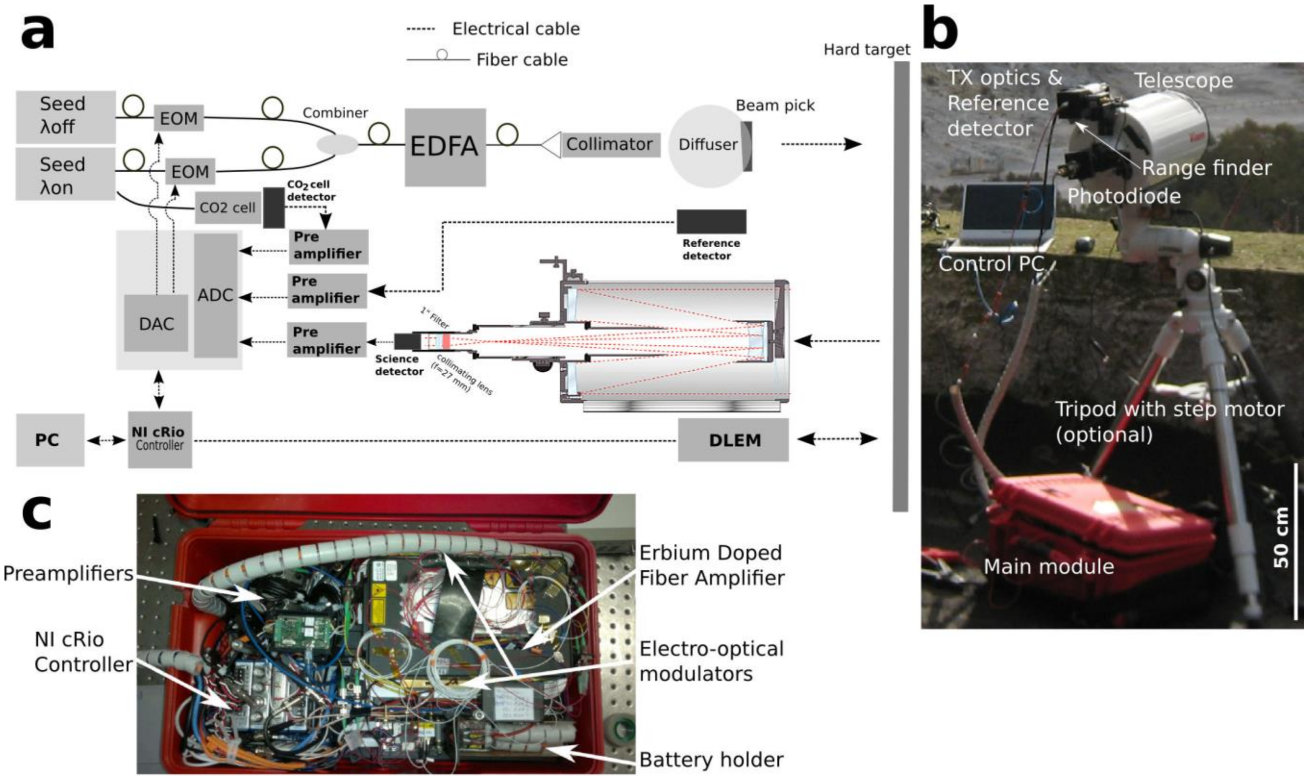

Supplementary Figure S1. Overview of the CO_2 DIAL as used for this experiment. (a) Scheme of the CO_2 DIAL. EOM: Electro-optical modulator, DLEM: range finder module, EDFA: Erbium doped fibre amplifier. ADC: analogue-to-digital converter, DAC: digital-to-analogue converter. The CO_2 cell is used to calibrate the seed laser wavelengths. To minimize hard target and turbulence related speckle noise the collimator used had a relatively high divergence of 1.7 mrad while the telescope field of view was 1.5 mrad. A wedged fused silica beam pick tapped off parts of the light for the reference power measurement. The detectors (InGaAs photodiodes) are thermally isolated from their surroundings to minimize drift in mechanical characteristics that may lead to non-stationary baseline drifts related to etalon effects. (b) Photo of the instrument with scale. (c) Overview of the components mounted on a bread board inside the main unit (red box). The CO_2 cell and the seed lasers are mounted on the bottom side of the breadboard (not visible).

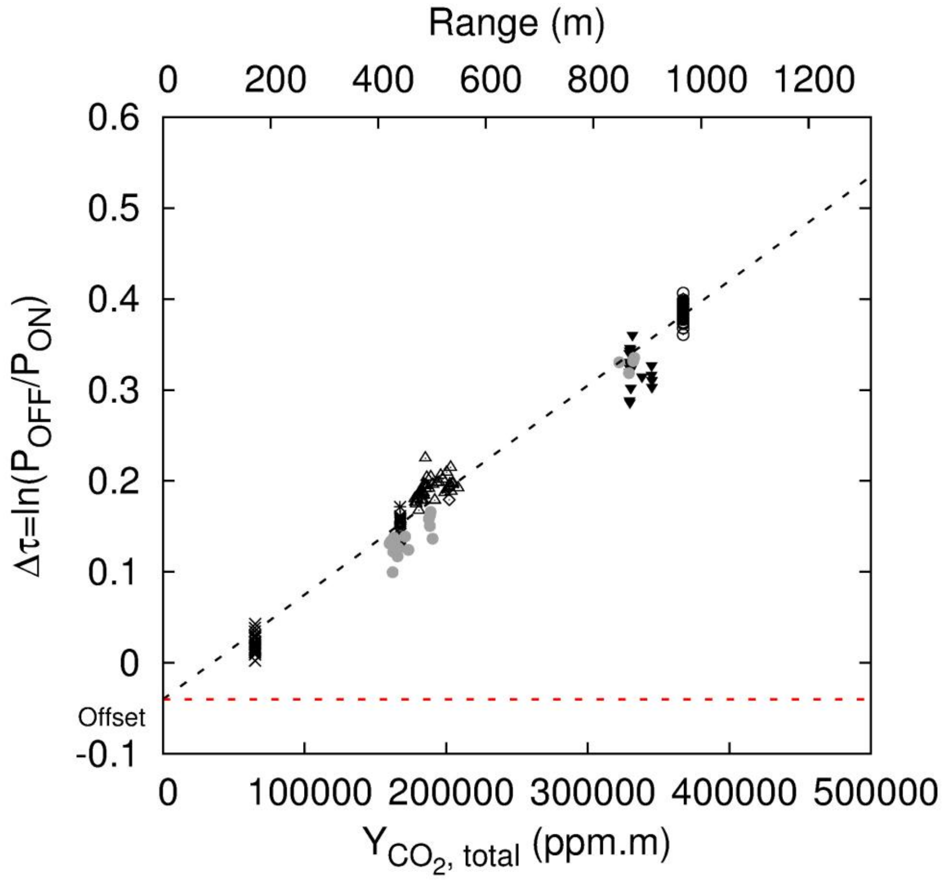

Supplementary Figure S2. Calibration data and linear fit. Shown are differential optical depths $\Delta\tau$ computed from the measured GR (grand ratios) versus the CO_2 path amount $Y_{CO_2,total}$, obtained for different hard target distances by multiplying the measured ranges (shown on top) with the known ambient CO_2 mixing ratio of 380 ppm. The symbols (cross, star, up- and down-pointing triangles and void circles) indicate different acquisitions. For instance, the down-pointing triangles (near 350000 ppm.m) are related to a small angle scan on 02/03/2016, while the crosses (near 70000 ppm.m) are related to a static measurement on 04/03/2016. Also shown is the fitted linear regression line. The instrumental offset b is -0.04 (equation 3 in Methods). The variance of the residuals χ^2 is 0.00031. For comparison, results of a measurement carried out 3 months earlier in another location with ambient CO_2 mixing ratio of 361 ppm are shown, indicated by the grey filled circles.

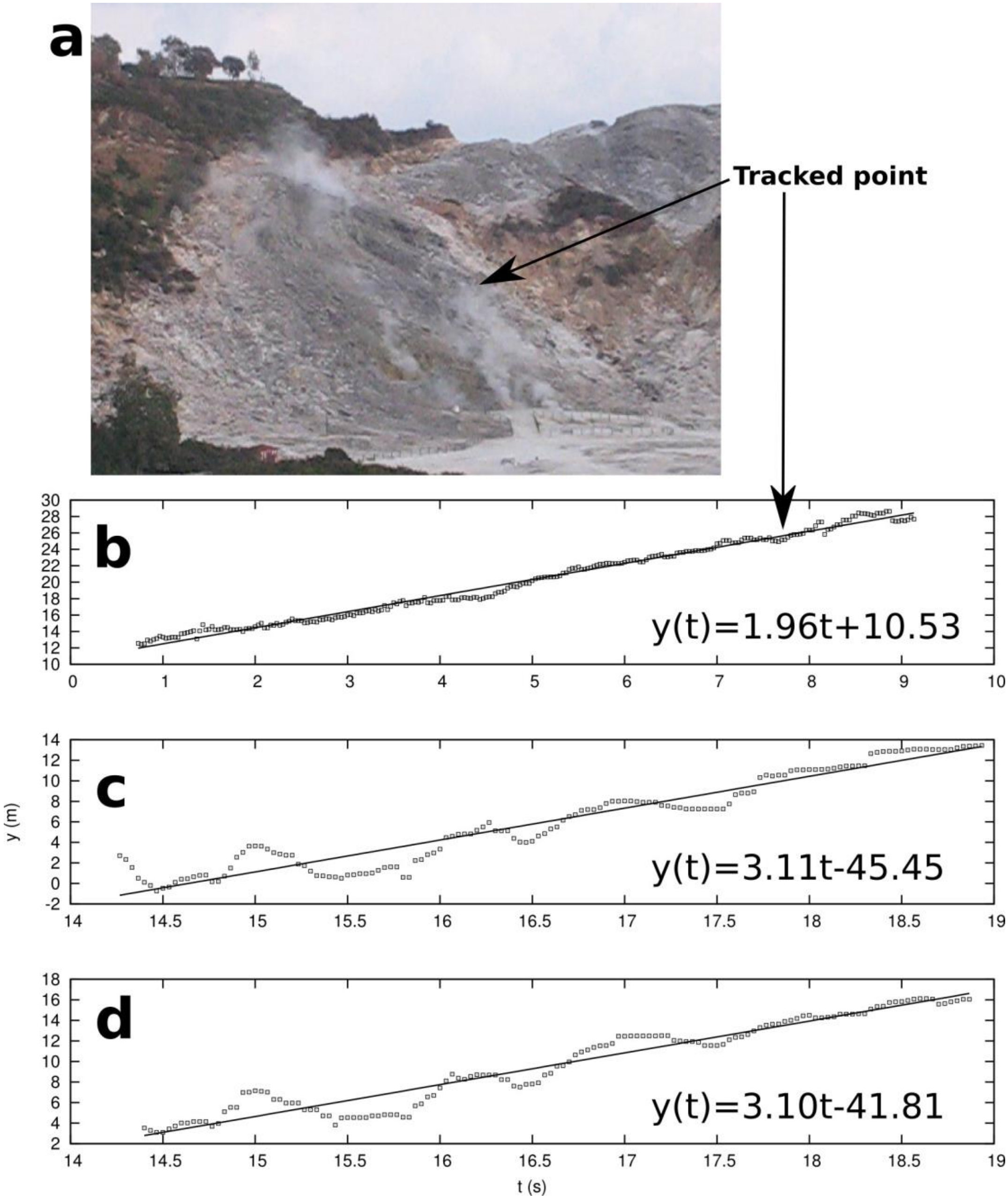

Supplementary Figure S3. Plume speed retrieval. (a) Snapshot of the video produced during the set of scans performed at Solfatara far field at the evening of 02/03/2016. One of the tracked pixels is marked. (b) Line fit. y-coordinate of the marked pixel versus time (seconds) and regression line used to estimate the vertical plume transport speed along with the fit parameters slope (vertical plume speed, in m/s) and y-axis intercept (in m). (c) y-track and regression line from Solfatara far field acquisition of the morning of 03/03/2016. The track is less steady, indicating an accelerated gas parcel and suggesting a more turbulent flow of gas than the evening before in (b). (d) y-track with regression line. The acquisition was the same as in (c), but corresponding to a parcel ~10 m next to that of (c). The track is very similar, and so are the estimated transport speeds, indicated by the regression line parameters.

Track	v_{pl} ± RMSE<br>Far field Solfatara<br>02/03/2016	Far field Solfatara<br>03/03/2016	Near field Solfatara<br>03/03/2016	Far field Pisciarelli<br>04/03/2016
1	1.96 ± 0.52	4.13 ± 0.59	0.66 ± 0.22	1.68 ± 0.38
2	1.09 ± 0.26	2.00 ± 0.63	1.43 ± 0.24	1.55 ± 1.46
3	1.53 ± 0.30	1.32 ± 0.53	1.10 ± 0.33	2.46 ± 1.11
4	2.01 ± 0.27	2.68 ± 0.57	2.24 ± 0.47	1.60 ± 0.73
5	0.76 ± 0.25	3.11 ± 1.21	1.67 ± 0.30	1.70 ± 0.50
6	1.86 ± 0.50	4.59 ± 1.01	2.38 ± 0.27	1.44 ± 0.49
7	1.51 ± 1.10	4.01 ± 0.87	2.43 ± 0.45	1.51 ± 1.10
8	1.54 ± 0.76	3.10 ± 0.87	1.51 ± 0.11	1.55 ± 0.76
Mean	1.53 ± 0.50	3.12 ± 0.81	1.68 ± 0.30	1.69 ± 0.82
Student's t Mean ± SD	1.53 ± 0.43	3.12 ± 1.11	1.68 ± 0.64	1.69 ± 0.33
$v_{pl} \pm \Delta v_{pl}$	**1.53 ± 0.50**	**3.12 ± 1.11**	**1.68 ± 0.64**	**1.69 ± 0.82**

Supplementary Table 1. Vertical plume transport speeds from various tracks. The units are in m/s for all values shown. For each track the root mean square error (RMSE) from regression line fit is shown. Student's t SD was computed from the speeds from the 8 tracks. Note that tracks 1 of Far field Solfatara 02/03/2016 and 5 and 8 of Far field Solfatara 03/03/2016 are shown in Fig. S3b, c and d, respectively. The final values of the vertical plume speed with their respective uncertainty used for the computation of CO_2 flux and flux error are shown in the bottommost row.
